# Deep learning in chest radiography: Detection of findings and presence of change

**DOI:** 10.1371/journal.pone.0204155

**Published:** 2018-10-04

**Authors:** Ramandeep Singh, Mannudeep K. Kalra, Chayanin Nitiwarangkul, John A. Patti, Fatemeh Homayounieh, Atul Padole, Pooja Rao, Preetham Putha, Victorine V. Muse, Amita Sharma, Subba R. Digumarthy

**Affiliations:** 1 Department of Radiology, Massachusetts General Hospital, Boston, Massachusetts, United States of America; 2 Harvard Medical School, Boston, Massachusetts, United States of America; 3 Division of Diagnostic Radiology, Department of Diagnostic and Therapeutic Radiology, Faculty of Medicine, Ramathibodi Hospital, Mahidol University, Bangkok, Thailand; 4 Qure.ai, 101 Raheja Titanium, Goregaon East, Mumbai, India; University of Texas MD Anderson Cancer Center, UNITED STATES

## Abstract

**Background:**

Deep learning (DL) based solutions have been proposed for interpretation of several imaging modalities including radiography, CT, and MR. For chest radiographs, DL algorithms have found success in the evaluation of abnormalities such as lung nodules, pulmonary tuberculosis, cystic fibrosis, pneumoconiosis, and location of peripherally inserted central catheters. Chest radiography represents the most commonly performed radiological test for a multitude of non-emergent and emergent clinical indications. This study aims to assess accuracy of deep learning (DL) algorithm for detection of abnormalities on routine frontal chest radiographs (CXR), and assessment of stability or change in findings over serial radiographs.

**Methods and findings:**

We processed 874 de-identified frontal CXR from 724 adult patients (> 18 years) with DL (Qure AI). Scores and prediction statistics from DL were generated and recorded for the presence of pulmonary opacities, pleural effusions, hilar prominence, and enlarged cardiac silhouette. To establish a standard of reference (SOR), two thoracic radiologists assessed all CXR for these abnormalities. Four other radiologists (test radiologists), unaware of SOR and DL findings, independently assessed the presence of radiographic abnormalities. A total 724 radiographs were assessed for detection of findings. A subset of 150 radiographs with follow up examinations was used to asses change over time. Data were analyzed with receiver operating characteristics analyses and post-hoc power analysis.

**Results:**

About 42% (305/ 724) CXR had no findings according to SOR; single and multiple abnormalities were seen in 23% (168/724) and 35% (251/724) of CXR. There was no statistical difference between DL and SOR for all abnormalities (p = 0.2–0.8). The area under the curve (AUC) for DL and test radiologists ranged between 0.837–0.929 and 0.693–0.923, respectively. DL had lowest AUC (0.758) for assessing changes in pulmonary opacities over follow up CXR. Presence of chest wall implanted devices negatively affected the accuracy of DL algorithm for evaluation of pulmonary and hilar abnormalities.

**Conclusions:**

DL algorithm can aid in interpretation of CXR findings and their stability over follow up CXR. However, in its present version, it is unlikely to replace radiologists due to its limited specificity for categorizing specific findings.

## Introduction

In 2010, approximately 183 million radiographic procedures were performed in the United States on 15,900 conventional and digital radiography units. Chest radiographs (CXR) represent close to half of the radiographs in the United States (44%). Most radiographs were acquired in outpatient clinics (48%) followed by hospital-based radiography [1]. An estimated annual growth rate of about 5.5% per year has been reported in prior surveys on radiography. On the one hand, there is tremendous volume and resultant burden, on the other hand, several past studies have reported challenges and inaccuracies associated with radiographic interpretation [2–4]. The Centers for Medicare and Medicaid Services (CMS) announced a 7% decrease in reimbursement for computed radiography from calendar year 2018 to address the issue of rising healthcare expenses.

Deep learning (DL) algorithms have been proposed as a solution to expedite, automate, and improve the interpretation of several imaging examinations including CXR. Prior studies have reported encouraging results of various DL algorithms for assessment of specific conditions such as pulmonary tuberculosis, cystic fibrosis, lines and tubes (position of peripherally inserted central catheters and endotracheal tubes), pneumoconiosis and lung nodules on CXR [5–16]. Another DL algorithm, now a commercially available application, subtracts ribs from single energy CXR to aid and expedite their interpretation by the radiologists.

To support and encourage research and development of DL in imaging, the National Institutes of Health (NIH) has released more than 100,000 deidentified CXR for free and open access [17]. We used these deidentified CXR datasets to assess the accuracy of a commercial deep learning (DL) algorithm for detection of abnormalities and to assess change in findings over serial radiographs.

## Methods and materials

Our retrospective study was performed on de-identified NIH image data of adult frontal CXR. Institutional review board (IRB) approval was waived.

### CXR data

Publicly available, de-identified ChestX-ray8 database (images and data entry file) was downloaded from the National Institute of Health website (https://nihcc.app.box.com/v/ChestXray-NIHCC accessed on January 30, 2018). This database contains labels for CXR based on presence and absence of 14 radiographic abnormalities. Several subjects have more than one CXR, which enable evaluation of stability or change in abnormalities over serial radiographs.

All CXR were selected randomly from the ChestX-ray8 datasheets (Microsoft EXCEL, Microsoft Inc., Redmond, Wash.) without looking at the accompanying CXR to ensure unbiased inclusion of CXR in the study. None of the standards of reference or test radiologists were part of the selection process.

Of the total 874 CXR included in the study, there were 574 single CXR (one CXR from each patient) from 574 patients and 300 CXR (two CXR from each patient) from the remaining 150 patients. Data labels of mass, nodules, pneumonia, atelectasis, or infiltration were grouped as pulmonary opacity; 374 CXR were selected from this group. Additional CXR were selected with data labels of effusion (n = 75), enlarged cardiac silhouette (n = 75), and those without any assessed findings (n = 200). Next, we identified CXR from 150 patients with (n = 100 patients) and without (n = 50 patients) any change in radiographic findings between the baseline and follow up CXR.

We excluded the lateral radiographs, oblique views, status post total pneumonectomy, and patients with a metal prosthesis, since the applied DL algorithm has not been trained for these views and situations. The final dataset included 874 CXR belonging to 724 patients (394M;330F) patients with mean age 54 ± 16 years. The radiographs in the ChestX-ray8 database are provided in PNG file format with 1024 x 1024 size and 8 bits gray-scale values.

### DL algorithm

The DL algorithm (Qure AI) assessed in our study has been installed in hospitals in India. The algorithm does not have the United States Food and Drug Administration (FDA) approval at the time of writing this manuscript.

The DL algorithm is based on a set of convolutional neural networks (CNNs), each trained to identify a specific abnormality on frontal CXR. A dataset of 1,150,084 CXR and the corresponding radiology reports from various centers in India were used to develop the algorithm. Natural language processing algorithms were used to parse unstructured radiology reports and extract information about the presence of abnormalities in the chest X-ray. These extracted findings were used as labels when training the CNNs. Individual networks were trained to identify normal radiographs as well as those with the following findings: blunting of costophrenic angle, pleural effusion, pulmonary opacity, consolidation, cavitation, emphysema, interstitial fibrosis, parenchymal calcification, enlarged cardiac silhouette, hilar prominence, and degenerative changes in the thoracic spine.

During the algorithm development process, radiographs were resized to a fixed size and normalized to reduce source dependent variance. The CNNs were then trained to detect abnormal findings on the resized radiographs. The network architectures, modified versions of either densenets [18] or resnets [19] were pre-trained on the task of separating CXR from radiographs of other body regions. For each abnormality, multiple models, both densenets, and resnets were trained with different parameters and augmentations. A majority ensembling scheme combines the predictions from these selected models and to decide the presence/absence of an abnormality. Algorithms were validated using an independent validation dataset of 93,972 frontal CXR.

**Fig 1:**
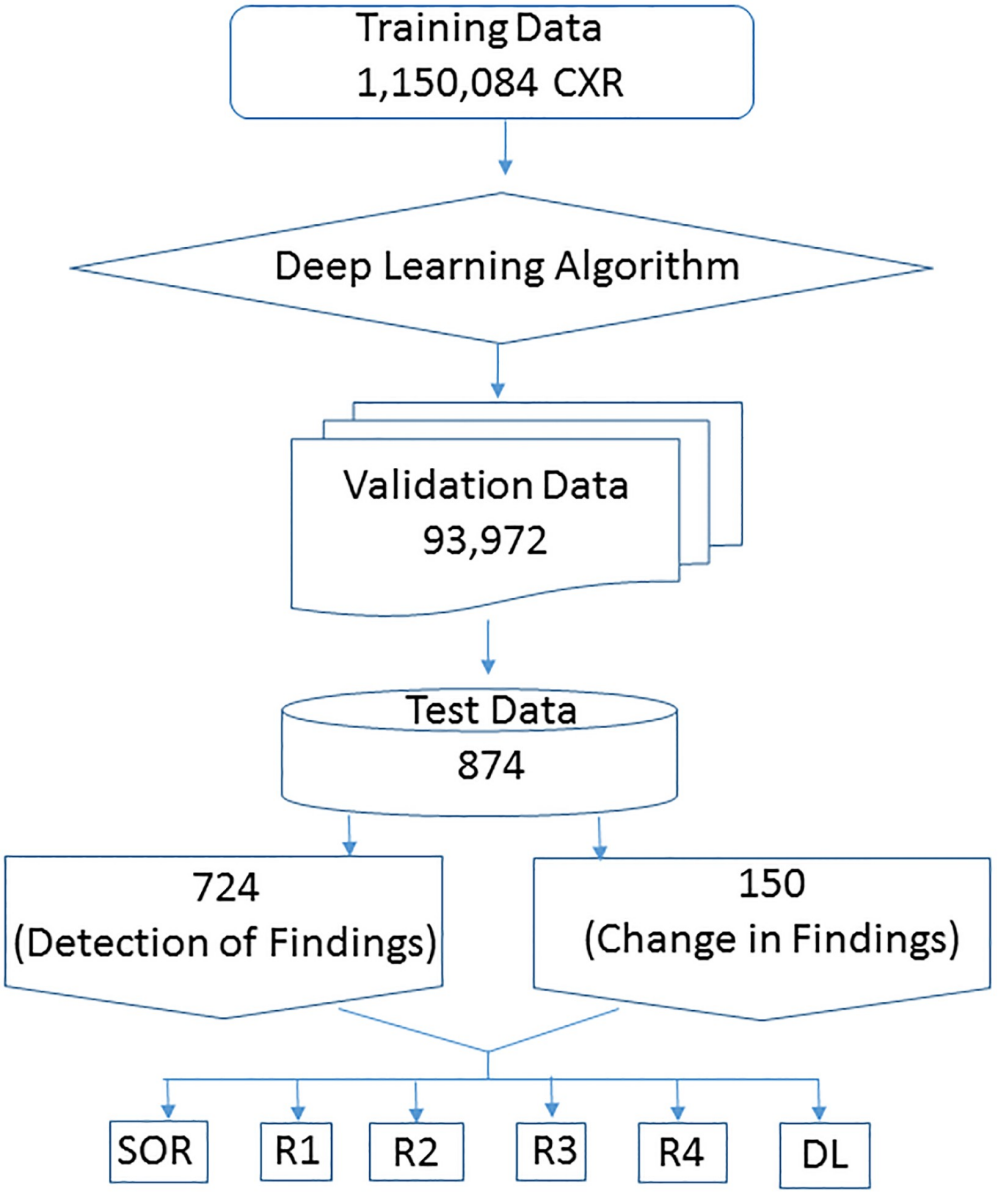
Flow-chart diagram illustrating development, validation, and testing of DL algorithm.

Specific abnormalities (cavitation, emphysema, interstitial fibrosis) were excluded from our study due to lower representation and reporting heterogeneity. The randomly selected 874 CXR’s in our study was neither used for training or validation of the algorithm Fig 1. For these CXR’s, the algorithm provided probability statistics in percentage likelihood for the presence of findings on a continuous scale (values between 0 to 1). Additional separate scores for predictions percentage, (0 for value < 0.5; and 1 for ≥ 0.5) were generated. Heat maps with annotated findings were displayed on duplicate copies of the CXR. The following four findings were assessed with the algorithm: pulmonary opacities, pleural effusions, hilar prominence, and enlarged cardiac silhouette.

### Standard of reference (SOR) and test radiologists

To establish the SOR, two experienced, fellowship-trained, thoracic subspecialty radiologists (SD with 16 years of subspecialty experience; MK with 12 years of subspecialty experience) assessed all 874 CXR in consensus for absence (score 0) or presence (score 1) of pulmonary opacities, pleural effusions, hilar prominence, and enlarged cardiac silhouette. SOR radiologists also recorded the presence of any lines, tubes and drains projecting over the CXR.

Separately, four thoracic subspecialty radiologists (JP with 35 years of experience; CN with 5 years of experience; AS with 25 years of experience and VM with 30 years of experience) served as test radiologists for the study. The test radiologists independently evaluated the presence of the previously described four findings in the 724 CXR on the same two-point scale (absence of a finding, score 0; presence, score 1). The test radiologists were unaware of the DL and SOR findings. A total of 150 serial CXRs were assessed for follow up of findings by SOR and test radiologists. Microsoft EXCEL worksheets were used for data entry and analysis of the findings.

### Statistical analysis

Microsoft EXCEL and SPSS statistical software (IBM SPSS Statistics, Armonk, NY) were used for data analysis. Receiver operating characteristics (ROC) analyses were performed to determine the separate area under the curve (AUC) for the DL algorithm and the four test radiologists against interpretation of the same findings by the SOR. Pair-wise two tailed t-tests (Microsoft EXCEL) were performed to compare detection of four radiographic abnormalities (pulmonary opacities, hilar prominence, pleural effusion, and enlarged cardiac silhouette) between the SOR and the DL algorithm as well as between the SOR and the four test radiologists. A p-value of less than 0.05 was deemed as statistically significant.

A post-hoc analysis (http://clincalc.com/stats/Power.aspx) was performed to determine if the number of radiographs with and without abovementioned abnormalities were adequate for assessing the DL algorithm.

## Results

### Detection of findings

**Fig 2:**
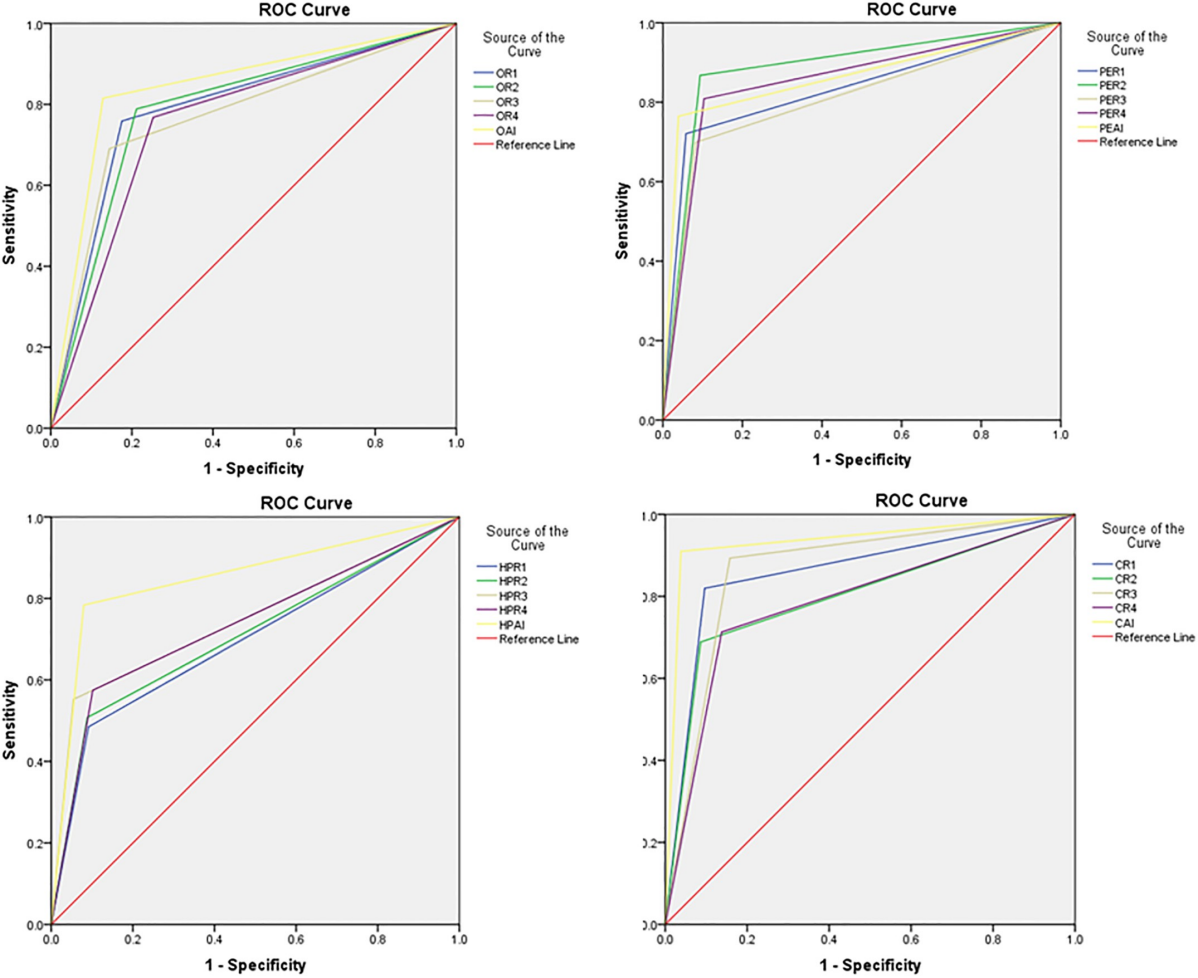
ROC curves for the DL algorithm and the four test radiologists (R1, R2, R3 and R4) in for pulmonary opacities (O), pleural effusion (PE), hilar prominence (HP) and enlarged cardiac silhouette (C).

**Fig 3:**
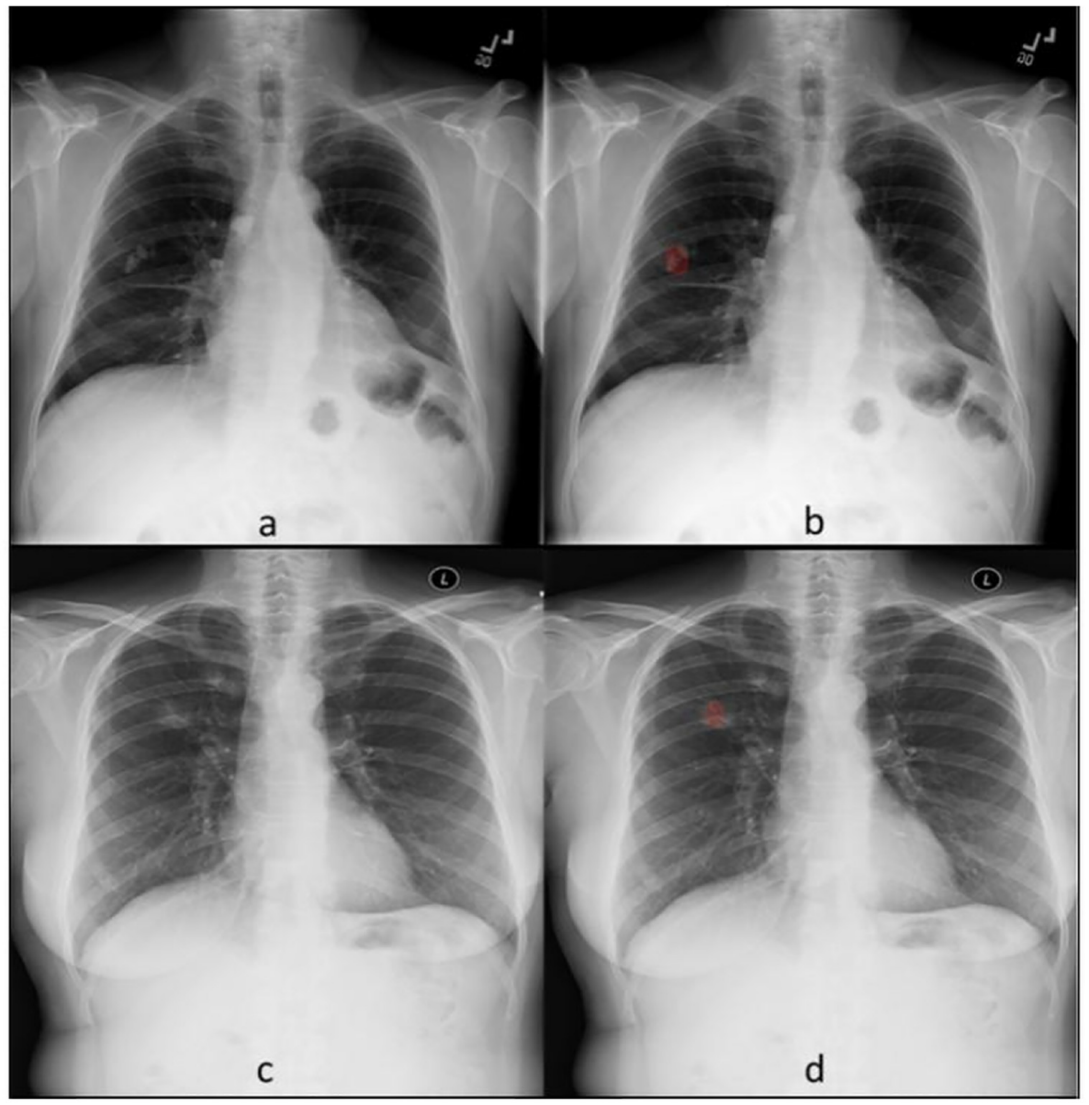
True positive pulmonary opacities. Frontal CXR belonging to two separate patients. Unprocessed CXR (a, c) demonstrate nodular opacities in the right lung. The corresponding heat maps from DL algorithm (b, d) accurately detected and annotated (in red) these abnormalities.

About 42% (303/724) with a single CXR had no pulmonary opacities, pleural effusion, hilar prominence, and enlarged cardiac silhouette (absence of labeled findings). Single and multiple findings were seen in 23% (168/724) and 35% (251/724) of CXR, respectively. Distribution of specific findings was: pulmonary opacities (336/724, 46%), pleural effusion (136/724, 19%), hilar prominence (134/724, 19%), and enlarged cardiac silhouette (122/724, 17%). The AUC for detection of CXR findings with the DL algorithm and the four test radiologists are summarized in Table 1. The AUC are shown in Fig 2. DL generated heat maps for detection of findings are as shown in Fig 3.

**Table 1:** Summary of AUC for detection of radiographic abnormalities in CXR.

	Detection of findings				
Attribute	DL	R1	R2	R3	R4
Enlarged cardiac silhouette	0.936 (0.905–0.967)	0.862 (0.820–0.904)	0.801 (0.750–0.852)	0.868 (0.832–0.904)	0.788 (0.738–0.837)
Pleural effusion	0.863 (0.819–0.906)	0.831 (0.785–0.878)	0.887 (0.851–0.923)	0.808 (0.761–0.856)	0.853 (0.812–0.894)
Pulmonary opacity	0.843 (0.812–0.874)	0.792 (0.757–0.826)	0.789 (0.754–0.823)	0.773 (0.737–0.809)	0.758 (0.722–0.794)
Hilar prominence	0.852 (0.809–0.895)	0.697 (0.641–0.752)	0.710 (0.654–0.765)	0.749 (0.695–0.803)	0.736 (0.683–0.790)

The AUC values for DL algorithm (DL) and the test radiologists (R1, R2, R3 and R4). The numbers in parenthesis represent AUC with 95% confidence interval.

**Fig 4:**
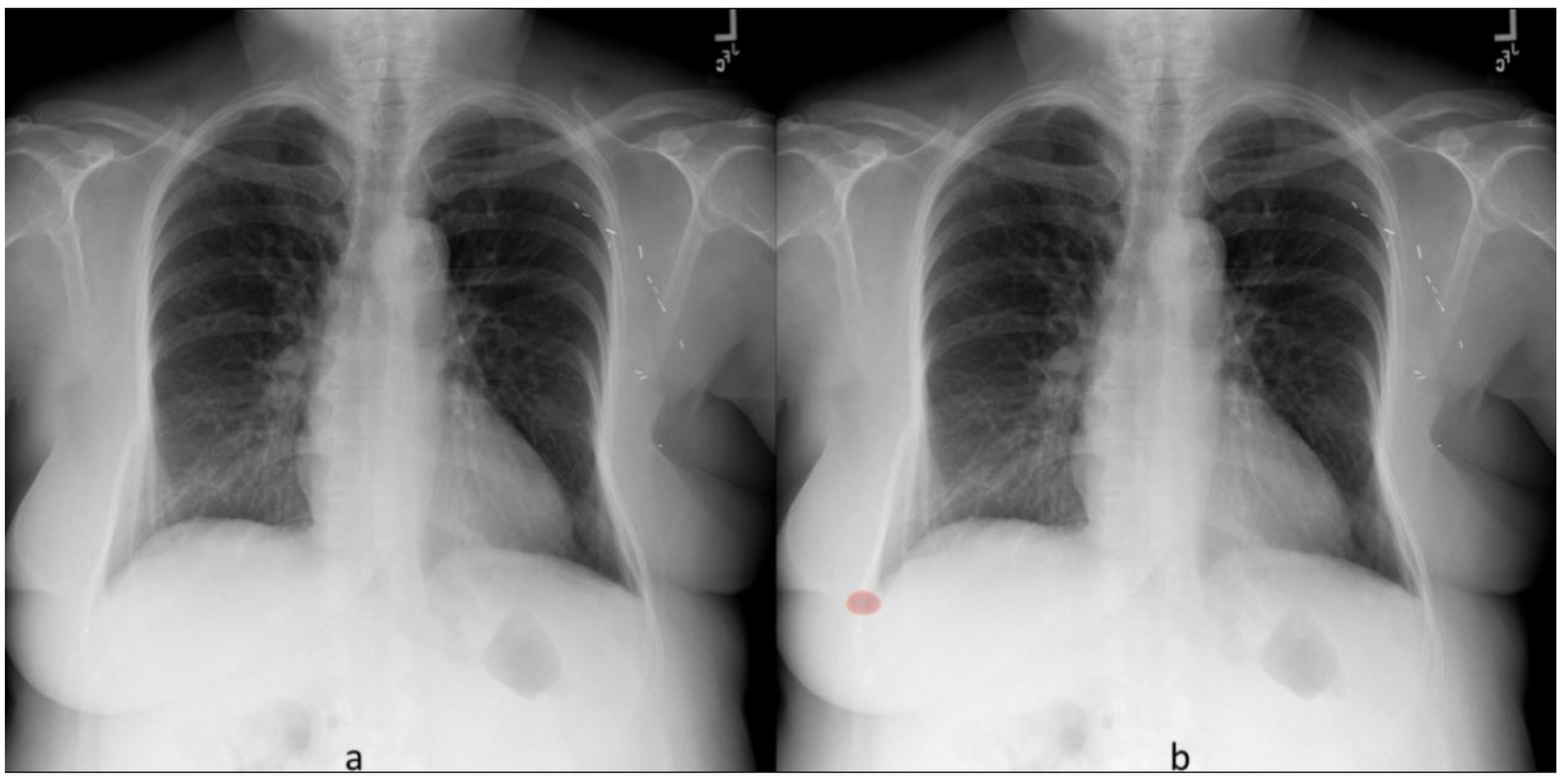
Frontal chest radiograph (a) without radiographic abnormality. The DL algorithm generated heat map (b) labels false positive pleural effusion on the right side.

The DL algorithm missed pleural effusions, hilar prominence, pulmonary opacities and cardiomegaly in 32, 29, 62 and 11 CXR, respectively. DL also had false positive pleural effusion (23 CXR), hilar prominence (47 CXR), pulmonary opacity (50 CXR) and enlarged cardiac silhouette (23 CXR). There was no statistical difference between DL and SOR for all abnormalities (p = 0.2–0.5). Frontal radiograph image with heat map for false positive detection of pleural effusion is shown in Fig 4.

The four test radiologists did not identify the following findings when compared to the SOR: pleural effusion (on 38, 18, 41, 26 CXR), enlarged cardiac silhouette (22, 38, 13, 35), hilar prominence (69, 66, 60, 57) and pulmonary opacities (81, 71, 104, 78). The test radiologists called false positive enlarged cardiac silhouette (on 58, 52, 95, 83 CXR), pulmonary opacities (68, 82, 56, 98), pleural effusion (34, 55, 48, 61) and hilar prominence (54, 52, 32, 60). There was a significant statistical difference in radiographic findings for enlarged cardiac silhouette between SOR and R1 (p = 0.02), SOR and R3 (p<0.05) and SOR and R4 (p = 0.002). There was statistically significant difference between SOR and R3 (p = 0.01) for hilar prominence.

**Fig 5:**
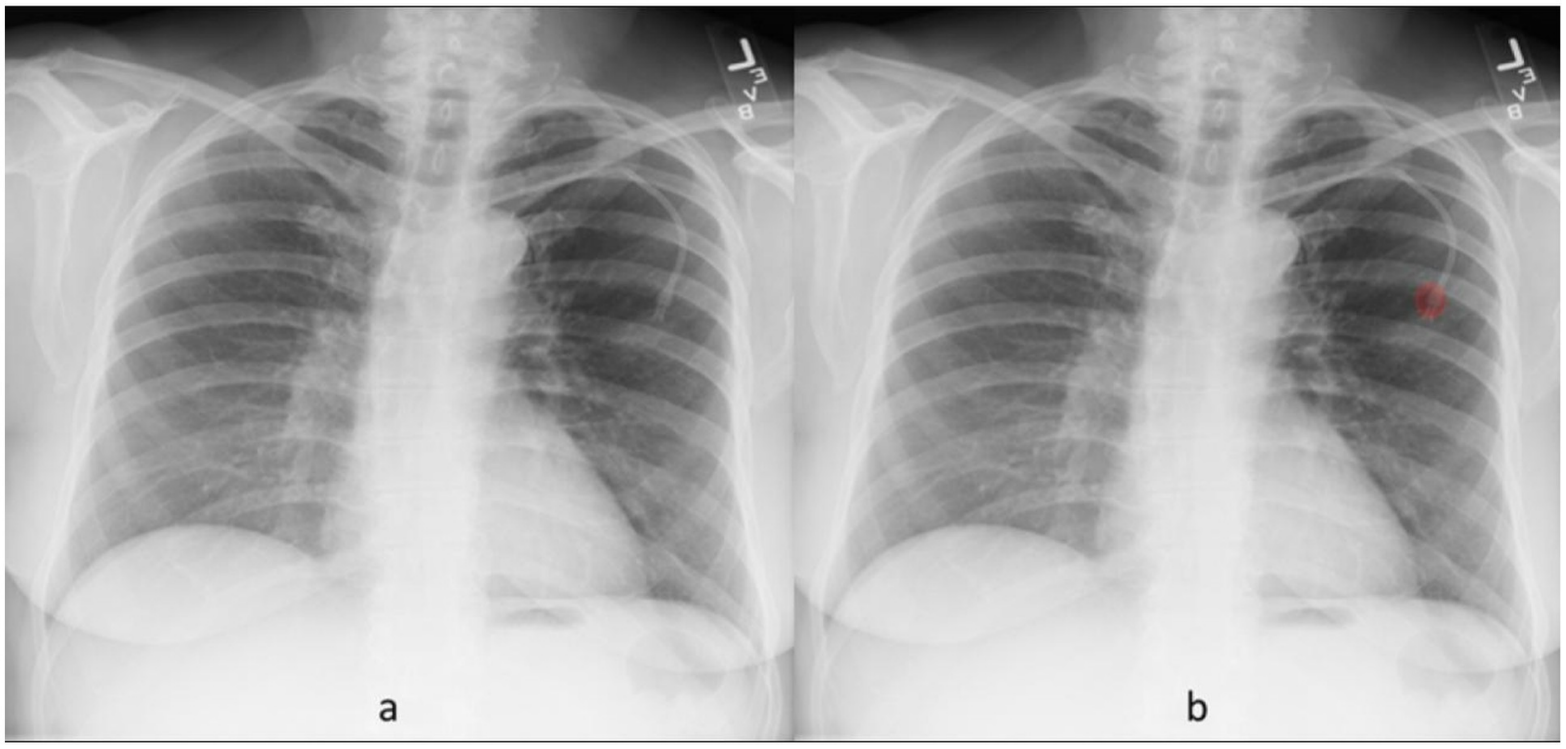
Implanted Port catheter projects in the left mid lung zone (a). Heat map from DL algorithm misinterpreted the port catheter as a focal pulmonary opacity. This is apparent on the accompanying heat map image (b).

In the included CXR, there were port catheters (62/113, 55%), peripherally inserted central catheter or PICC lines (16/113, 14%), intercostal chest tubes (14/113, 12%), generators for pacemaker or defibrillator devices (14/113, 12%), and chest wall ECG leads (7/113, 6%). Implanted port catheters and generators of pacemaker or defibrillator interfered with the performance of the DL algorithm and resulted in false positive pulmonary opacity in 15% (17/113) of CXR with such devices. Some implanted port catheters (14/62, 23%), intercostal chest tubes (2/14, 14%), and ECG leads (1/7, 14%) were interpreted as false positives for pulmonary opacities (as depicted in Fig 5).

### Changes in findings

A pair of radiographs was available for 150 within the subset of 724 CXR. The pair of 150 radiographs (150x 2, 300 radiographs) were used to assess change.

In the baseline CXR, SOR reported pulmonary opacities (58/150 CXR, 39%), pleural effusions (43/150 CXR, 29%), hilar prominence (29/150 CXR, 19%), and enlarged cardiac silhouette (28/150 CXR, 19%). On the follow up CXR, there were pulmonary opacities (62/150, 41%), pleural effusions (42/150, 28%), hilar prominence (31/150, 21%), and enlarged cardiac silhouette (25/150, 17%) (P = 0.001–0.02).

**Fig 6:**
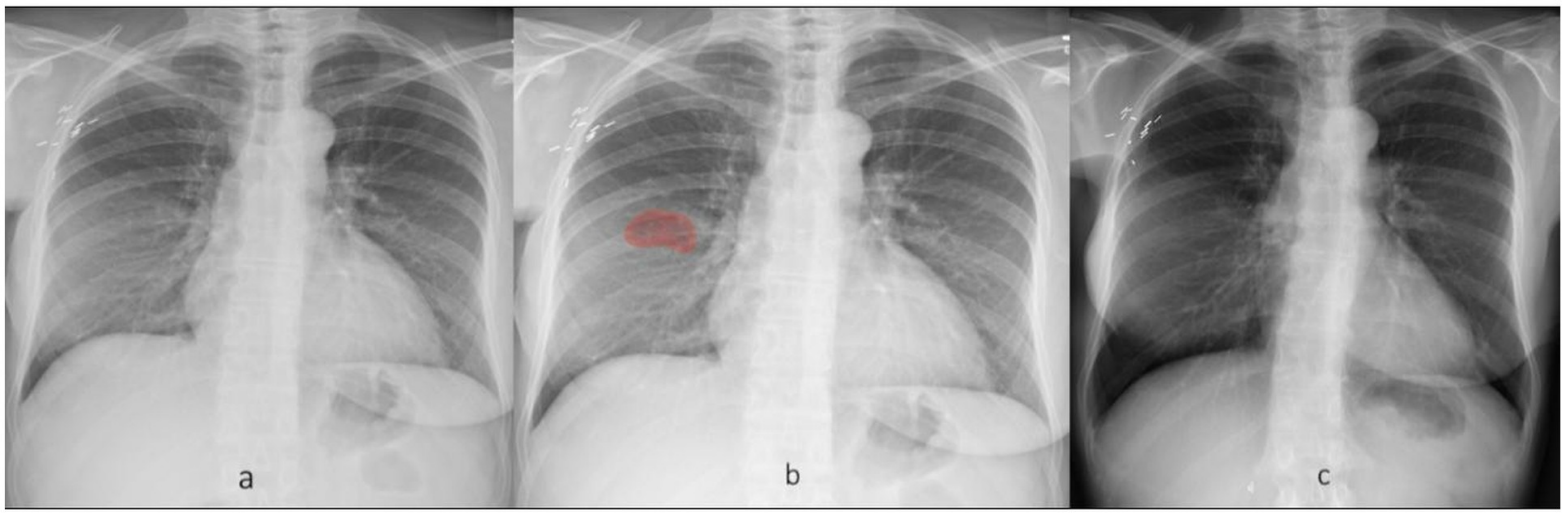
Frontal CXR demonstrates a subtle patchy opacity in right mid zone (a), which is annotated (in red) on the heat map generated from DL algorithm (b). On subsequent follow-up CXR (c), the heat map did not mark any abnormality (resolution).

On the follow-up CXR, SOR found following changes compared to the baseline CXR: pulmonary opacities (33/150, 22%), pleural effusion (21/150, 14%), enlarged cardiac silhouette (13/150, 9%), and hilar prominence (8/150, 5%). The corresponding changes for DL were 35% (53/150), 23% (34/150), 23% (34/150), and 23% (35/150). For the test radiologists, the corresponding changes in findings were 22–30% (33-46/150), 13–22% (20-33/150), 8–13% (12-20/150), and 5–12% (7-18/150), respectively. The AUC for detecting the change in CXR findings with the DL algorithm and for the four test radiologists are summarized in Table 2. The heat maps are as shown in Fig 6.

**Table 2:** Summary of AUC for change in abnormalities over follow-up CXR.

	Change in findings				
Attribute	DL	R1	R2	R3	R4
Enlarged cardiac silhouette	0.925 (0.837–1.000)	0.680 (0.505–0.855)	0.793 (0.628–0.958)	0.680 (0.505–0.855)	0.744 (0.572–0.916)
Pleural effusion	0.782 (0.657–0.907)	0.752 (0.617–0.886)	0.670 (0.531–0.809)	0.808 (0.696–0.921)	0.849 (0.746–0.952)
Pulmonary opacity	0.687 (0.572–0.802)	0.702 (0.591–0.813)	0.778 (0.675–0.881)	0.833 (0.750–0.917)	0.783 (0.687–0.879)
Hilar prominence	0.735 (0.533–0.937)	0.569 (0.349–0.789)	0.607 (0.379–0.836)	0.579 (0.357–0.831)	0.770 (0.565–0.975)

The AUC for the DL algorithm and the test radiologists (R1, R2, R3 and R4). The numbers in parenthesis represent AUC with 95% confidence interval.

### Post-hoc power calculation

Post-hoc power analysis revealed that (http://clincalc.com/stats/Power.aspx) study was adequately powered for detecting up to 95% of the assessed abnormalities in 421 abnormal CXR and up to 1% false positive abnormalities in 303 CXR without any assessed abnormalities with a type 1 error rate of 0.01.

## Discussion

### Detection of CXR findings

The overall accuracy of DL algorithm was better or equal to test radiologists with different levels of experience. DL algorithm had similar accuracy for detection of enlarged cardiac silhouette, pleural effusion and pulmonary opacities in CXR. The DL algorithm, as well as the test radiologists, had the highest accuracy for assessing enlarged cardiac silhouette. Prior studies from Wang et al. (AUC 0.807) [17], Yao et al. (AUC 0.0904) [20] and Rajpurkar (AUC 0.9248) [21] have also reported a higher accuracy of DL algorithms for detection of cardiomegaly. In line with the radiology reports of CXR at our institution, we chose “enlarged cardiac silhouette” as a descriptor for cardiomegaly used in other previously published studies [17, 20, 21].

The accuracy of our DL algorithm for detecting pleural effusions (AUC 0.872) is better than the accuracy reported by Wang et al. (AUC 0.784) and nearly identical to accuracies reported by Yao et al. (AUC 0.859) and Rajpurkar et al. (AUC 0.8638) [17, 20, 21]. These studies [17, 20, 21] used the same ChestX-ray8 datasets that were used in our study. The AUC of DL for detection of pleural effusions and pulmonary opacities in our study was lower than perfect accuracy (AUC 0.95–1.00) reported by Becker et al [22]. These differences can be attributed to variations in DL techniques, sample size, patient population, a gamut of radiographic findings, and quality of CXR between the two studies.

Detection of hilar prominence was more accurate with the DL algorithm than for either of the test radiologists. To our best knowledge, this finding has not been assessed in prior studies on DL algorithms for CXR interpretation. The higher performance of DL relative to the radiologists may be explained based on the relatively subjective nature of this finding compared to other findings. Conceivably, with different sets of SOR radiologists or test radiologists, the accuracy of hilar prominence might have been different. This implies that instead of radiologists, a better SOR for CXR is chest CT from the same patient since it can provide robust and objective verification of ground truth for complex radiographic findings such as hilar prominence that can be overcalled due to differences in techniques, patient rotation, and lung volumes.

Pulmonary opacities such as atelectasis, infiltration, pneumonia, consolidation, fibrosis, mass, and nodules were analyzed separately in prior publications [17, 20, 21]. The DL algorithm in our study was not trained to classify the opacities as pulmonary fibrosis, masses, and nodules, and thus these findings were not separately assessed in our study. Furthermore, atelectasis, infiltration, pneumonia, and consolidation can co-exist in the same anatomic region of the lung, and their radiographic appearance can be similar. Therefore, we assessed these findings jointly as pulmonary opacities. The accuracy of detection of pulmonary opacities in our study was not different from those reported in prior studies [17, 20, 21] for similar abnormalities.

### Change in CXR findings

Although substantially better than the four test radiologists for presence or lack of changes in pulmonary opacities, the accuracy of DL as noted from the ROC analyses (Table 2) was lowest when compared to the SOR. This may have been due to variations in radiographic technique, or patient-related factors (such as differences in inspiratory effort and patient rotation over serial radiographs) on the appearance of pulmonary opacities. To our best knowledge, prior studies have not assessed the accuracy of DL for change or stability in findings on serial CXR.

### Implications

Our study implies that DL based algorithm has high accuracy for detection of specific radiographic abnormalities such as pulmonary opacities, pleural effusions, enlarged cardiac silhouette, and hilar prominence. More work is needed to optimize the performance on follow up CXR, particularly for pulmonary opacities. The study also raises questions regarding the validity of radiologists as appropriate SOR given the subjectivity associated with detection and classification of radiographic findings. Differences between accuracy of test and SOR radiologists raise concern about the consistency and subjectivity of radiographic interpretation suggesting the need for a better SOR such as chest CT. Therefore, the results of our study should be interpreted with caution. However, substantially better sensitivity of CT compared to CXR will also confound Further improvements in the assessed DL algorithm are needed to classify pulmonary opacities and elucidate their anatomic location. DL algorithms for CXR interpretation should also detect, segment, and exclude implanted devices and external foreign objects projecting over the lung fields, which can otherwise result in false positive findings as noted in our study. Despite these limitations, our study adds to a growing body of evidence regarding the utility of DL algorithms for detection of findings on CXR.

There are additional limitations in our study. We did not perform pre-hoc power analysis to determine the number of CXR and test radiologists required to assess the performance of the DL algorithm. However, the number of CXR included in our study was higher than those used in prior studies and was found to be adequate for assessing the DL algorithm on post-hoc analysis. Another limitation of our study pertains to the combined evaluation of different types of pulmonary opacities rather than separate categories reported in prior studies [17, 20, 21]. Non-inclusion of specific CXR findings in the DL algorithm like the position of lines and tubes, pneumothorax, pulmonary nodules, masses, and fibrosis, was not possible as the algorithm was not specially trained for those findings. Although the DL algorithm provided prediction statistics in percentage likelihood of findings on a continuous scale (values between 0 to 1), we relied on the binary scores (that is, finding absent for prediction percentage < 0.5 and finding present for prediction percentage ≥ 0.5) as recommended by the vendor. It is possible that specificity and accuracy of DL findings may have been different with the use of cut-off values other than 0.5. There are however no published guidelines on the most appropriate cut-off values for DL algorithm and how such deviations would affect the performance of DL.

## Conclusions

In conclusion, a DL algorithm can aid in interpretation of CXR findings such as pulmonary opacities, hilar prominence, cardiomegaly and pleural effusion. It can also help in assessing change or stability of these findings on follow-up CXR. Though helpful in improving the accuracy of interpretation, the assessed DL algorithm is unlikely to replace radiologists due to limitations associated with the categorization of findings (such as pulmonary opacities) and lack of interpretation for specific findings (such as lines and tubes, pneumothorax, fibrosis, pulmonary nodules, and masses). However, DL algorithm can expedite image interpretation in emergent situations where a trained radiologist is either unavailable or overburdened in busy clinical practices. It may also serve as a second reader for radiologists to improve their accuracy.
